# A repeated cross-sectional study examining the school impact on child weight status

**DOI:** 10.1016/j.ypmed.2014.04.003

**Published:** 2014-07

**Authors:** Andrew James Williams, Katrina Mary Wyatt, Craig Anthony Williams, Stuart Logan, William E. Henley

**Affiliations:** a Institute of Health Research, University of Exeter Medical School (formerly Peninsula College of Medicine and Dentistry), Veysey Building, Salmon Pool Lane, Exeter, Devon EX2 4SG, United Kingdom; b Children's Health and Exercise Research Centre, Sport and Health Sciences, College of Life and Environmental Sciences, University of Exeter, St. Luke's Campus, Heavitree Road, Exeter, Devon EX1 2LU, United Kingdom

**Keywords:** BMI-SDS, body mass index standard deviation score, LSOA, lower super output area, NCMP, National Child Measurement Programme, League tables, Obesity, Education, Value-added

## Abstract

**Objective:**

The aim of this study is to examine whether there is a differential impact of primary schools upon children's weight status.

**Methods:**

A repeated cross-sectional study was undertaken using five years (2006/07–2010/11) of National Child Measurement Programme data, comprising 57,976 children (aged 4–5 (Reception) and 10–11 (Year 6) years) from 300 primary schools across Devon, England. Examining each year separately, the schools were ranked according to their observed and residual (having accounted for school and neighbourhood clustering and pupil ethnicity and socioeconomic status) school mean body mass index standard deviation score (BMI-SDS). Subtracting the Reception from the Year 6 mean residuals gave ‘value-added’ scores for each school which were also ranked. The rankings were compared within and across the years to assess consistency.

**Results:**

Although pupil BMI-SDS was high, > 97% of the variation in BMI-SDS was attributable to environments other than the school. The ‘value-added’ by each school was only poorly correlated with the observed and residual pupil BMI-SDS; but none of the rankings were consistent across the five years.

**Conclusion:**

The inconsistency of the rankings and the small variation in BMI-SDS at the level of the school suggests that there is no systematic differential impact of primary schools upon pupil weight status.

## Highlights

• < 3% of the variation in primary school pupil weight status is attributed to school.
• Rankings of schools based on pupil weight status were inconsistent across 5 years.
• League tables are inapt for identifying schools to target for obesity prevention.

## Introduction

Non-communicable diseases are now the leading cause of death world-wide (Beaglehole et al., 2011; General Assembly of the United Nations, 2011). Obesity as a risk factor for a number of non-communicable diseases has become a public health priority (Beaglehole et al., 2011). The rising prevalence of obesity, coupled with the realisation that several of the determinants of obesity originate in or before childhood, has led to many preventative efforts being concentrated on children (Butland et al., 2007; Procter, 2007). Moreover, schools, where children congregate to learn, eat, and share activities are readily accessible environments for prevention (Brown and Summerbell, 2009; Khambalia et al., 2012; Procter, 2007; Procter et al., 2008). Within England it has been observed that the prevalence of obesity doubles during the period of primary education (4–11 years of age), leading to questions about whether schools themselves are obesogenic environments (Ridler et al., 2009).

To date, no interventions which sought to affect the school environment or context have been found to have a lasting effect on the prevalence of obesity (Khambalia et al., 2012). Moreover, there is little empirical evidence of any impact of the school environment upon children's weight status (Bonell et al., 2013; Williams et al., 2012, 2013). One of the few papers to examine whether schools had an impact on children's weight status was produced by Procter et al. (2008) who hypothesised:

‘[t]hat by exploring differences between schools, we may be able to determine school factors that are, for better or worse, having an impact on children’s risks of obesity. At the same time, we may be able to highlight ‘hot’ and ‘cold’ spots of obesity so allowing better targeting of resources to those communities in greatest need.’

Procter et al. (2008) p.342.

To test this hypothesis Procter et al. (2008) employed a ‘value-added’ technique similar to those developed in economics and regularly used to assess the educational impact of schools (Amrein-Beardsley, 2008; Rutter, 1979). In education, an individual's value-added score is the change in outcome (e.g. test score) during the period of their schooling. In order to compare school performance the individual scores are aggregated, and it becomes necessary to adjust for differences in school composition which could bias the scores (Amrein-Beardsley, 2008; Rutter, 1979). Procter et al. (2008) accounted for the ethnic and socioeconomic composition of 35 primary schools in Leeds, England, who were participating in the Trends study to rank schools according to their mean observed and expected residual pupil weight status and ‘value-added’ score. The authors found that there was little similarity between the ‘value-added’ and expected residual rankings and concluded that this lent credence to the hypothesis that differing school environments have differential impacts upon their pupils (Procter et al., 2008). As a result they suggested that obesity prevention efforts be targeted rather than population wide as ‘hot’ and ‘cold’ schools for obesity had been identifiable, and hence future research should focus on such schools. Acknowledging the fallibility of such ‘league tables’, Procter et al. (2008) also suggested that these analyses should be replicated across a number of years to test the validity of the findings (Goldstein and Spiegelhalter, 1996). This study evaluates and expands upon the technique proposed by Procter et al. (2008) using repeated cross-sectional data from a large routine data source (the National Child Measurement Programme (NCMP)) to examine the potential differential impact of primary schools on children's weight status.

## Methods

The English NCMP was introduced in 2005 to monitor progress towards a public service agreement to reduce the prevalence of obese primary school aged children (Dinsdale and Rutter, 2008; South East England Public Health Observatory, 2005). Unless individuals or schools are actively opted out, all Reception (4–5 year olds) and Year 6 (10–11 year olds) pupils in state maintained primary schools have their height and weight measured by a health professional (Dinsdale and Rutter, 2008). Five years of NCMP data (2006/07–2010/11, involving 57,976 pupils) from Devon local authority were used in this study. The child's gender and age at time of measurement collected within the NCMP were used to calculate their body mass index standard deviation score (BMI-SDS) using the United Kingdom 1990 reference population and the LMS method defined by Cole et al. (1995). The child's ethnicity (Department for Education classification), neighbourhood (Lower Super Output Area (LSOA)), school and year group were also recorded (The NHS Information Centre, 2012). Like Procter et al. (2008) we were able to link each child's LSOA to the Index of Multiple Deprivation as a measure of socioeconomic status (Department for Communities and Local Government, 2011). Prior to linking the 2010 Index of Multiple Deprivation to the NCMP data the score was nationally rescaled from 0 to 1 (normalised), to aid interpretation (Goldstein, 2003). The Department for Education ethnicity categories were collapsed into the following five categories to ensure that there were sufficient numbers in each category for analysis; White–British; Any other White background; Chinese, Asian or Asian British; Mixed/Dual background; and Any other ethnic group (including Black or Black British) (Department of Health, 2009). Procter et al. (2008) studied Year 4 (8–9 year olds) rather than Year 6 pupils alongside Reception pupils and used a binary ethnicity classification (south Asian or non-south Asian); otherwise the data sets are similar and both cross-sectional. Consequently, it was possible to apply the method proposed by Procter et al. (2008) within each of the five years of the NCMP data set as outlined below.

### Statistical analyses

In education, school-level value-added scores are used as comparable measures of the average improvement in pupil attainment while attending the school. To ensure fair comparisons of different schools, it is important to adjust for differences in school composition. The following steps were taken to apply ‘value-added’ methods to pupil weight status.

#### Step 1

*Rank schools according to their observed mean BMI-SDS (Observed ranking)*. Following Procter et al. (2008) both year groups were combined to calculate each school's mean BMI-SDS. The ranking of schools based upon their observed mean BMI-SDS was recorded, giving a rank of the schools with lowest to highest mean pupil weight status. This Observed ranking is not a reflection of school effect on weight status as differences in mean BMI-SDS could relate to differences in school composition (e.g. demographics) or be a reflection of the pre-school (baseline) pupil weight status.

#### Step 2

*Rank schools according to how much their observed mean BMI-SDS differed from the expected (‘Expected’ ranking).* The next step was to adjust the data to determine the extent to which the school's mean pupil weight status differs from that expected. As ethnicity and socioeconomic status are widely recognised determinants of obesity, these were the pupil characteristics used to calculate the expected mean pupil BMI-SDS (Butland et al., 2007). Two-level models, cross-classified by school and neighbourhood (LSOA) in order to account for the fact that children from the same neighbourhood may not attend the same school and vice versa, were used to calculate the expected mean pupil BMI-SDS (Procter et al., 2008). In order to test the need for cross-classification by neighbourhood (LSOA), models with and without neighbourhood cross-classification were tested at this stage. The ranking of schools based upon the extent to which the observed mean BMI-SDS differed from the expected mean BMI-SDS was recorded (Expected residuals). Schools with observed mean pupil weight status which is markedly different from that expected (i.e. high or low residuals) may represent hot and cold spots of obesity.

#### Step 3

Calculate and rank schools according to a ‘value-added’ score (‘Value-added’ ranking)

The ‘Expected’ ranking gives a measure of the impact of the school, but does not account for pre-school weight status. As the data were cross-sectional, differences within-pupils could not be calculated. Instead, differences between year groups of pupils were calculated through an identical process to that used by Procter et al. (2008). As Reception is the first year of schooling Reception pupils are relatively unexposed to the school environment and context compared with pupils in Year 6, and therefore the Reception pupil weight status was conceptualised as the pre-school weight status. The expected residuals for Reception and Year 6 pupils were calculated separately using the same multilevel model as in Step 2. The difference between these two sets of expected residuals gave a measure (score) of the average ‘value-added’ to the pupil BMI-SDS by the school, the ranking of which was recorded.

#### Step 4

*Compare the Observed, ‘Expected’ and ‘Value-added’ rankings*. Primarily Lin's concordance correlation coefficients (*ρ*_c_) (Lin, 1989, 2000; Steichen and Cox, 2002) were used to quantify the agreement between pairs of rankings within each of the five years. Pearson's correlation coefficients (*r*) were calculated alongside the concordance values, and the rankings were visualised in caterpillar plots; these additional analyses are reposted in the supplementary material.

#### Step 5

Compare stability of the rankings across the five years (2006/07–2010/11)

Within each ranking, concordance correlation coefficients were calculated comparing the agreement between each of the five years of rankings. As with the previous step Pearson's correlation coefficients and caterpillar plots are reported as supplementary material. Tracking coefficients (kappa) were calculated to explore the extent to which schools maintained approximately the same rankings across the five years. In order to quantify approximate positions, the rankings of schools were split into quintiles each year, prior to the calculation of the tracking coefficients. There was no comparison between the three types of ranking in this step.

The analysis was undertaken in Stata 11 (StataCorp, 2009) with the models estimated using numerical integration with seven quadrature points and restricted maximum likelihood estimation. Due to a sparse matrix in 2010/11 it was necessary to estimate the cross-classified model in R (R Development Core Team, 2011) using lme4 (Bates et al., 2011) and then transfer the results back into Stata.

## Results

The sample characteristics and the results of the cross-classified models fitted to calculate each school's expected mean BMI-SDS are shown in Table 1. Only a small proportion of the variation in pupil BMI-SDS was attributed to either the school or the neighbourhood in the null models (intraclass correlation coefficients < 0.03). There was a significant association between socioeconomic status and BMI-SDS, with the regression coefficient for the Index of Multiple Deprivation calculated to show the mean difference in BMI-SDS between the most and least deprived LSOAs in England, based upon the trend in Devon. A subsample comprising 10 schools, approximately equally distributed across the 2006/07 Observed ranking, were selected in order that the change of rankings in some individual (anonymised) schools could be observed (Table 2). The data presented in Table 2 clearly demonstrate that whilst within each year the Observed and ‘Expected’ rankings of schools are similar, the ‘Value-added’ rankings are considerably different. Furthermore, across the five years there was substantial movement in school position in each of the three rankings. The levels of agreement (concordance (*ρ*_c_ values)) between each of the three rankings within each year are presented in Table 3. These values confirm the observations from Table 2: within each year the agreement between the Observed and ‘Expected’ rankings were high (*ρ*_c_ ~ 0.9), whereas the concordances with the ‘Value-added’ rankings are much lower (*ρ*_c_ < 0.3). The equivalent Pearson's correlation coefficients are reported in Table S1 and the caterpillar plots in Fig. S1 of the supplementary material, which further confirm the above findings.

The results of the analyses testing how stable the rankings were across the five years are presented in Table 4. These show that within each individual ranking (Observed, ‘Expected’ and ‘Value-added’) the concordance values were small (*ρ_c_* < 0.25), demonstrating that across the years the rankings varied considerably; notably, the level of agreement across the ‘Value-added’ rankings was even smaller (*ρ*_c_ < 0.1). These results demonstrate the lack of consistency in any of the rankings across the five years. The equivalent Pearson's correlation coefficients are reported in Table S2 and caterpillar plots in Fig. S2; further supporting the findings presented in Table 4. The kappa values, which show the extent to which schools maintained approximately the same rankings across the five years were, 0.06 (*p* < 0.0001), 0.06 (*p* < 0.0001) and 0.05 (*p* < 0.0001) for the Observed, ‘Expected’ and ‘Value-added’ rankings respectively. Similar to Procter et al. (2008), it was found that repeating all the analyses without cross-classification by neighbourhood made very little difference to the results (Tables S3–S8, and Figs. S3 and S4).

## Discussion

Using a large sample of data from the NCMP and a repeated cross-sectional design, this study has examined the possibility of a ‘school effect’ on pupil weight status. The ranking of schools based on the mean ‘value-added’ to pupil weight status, adjusted for individual ethnicity and socioeconomic status, produced rankings which had little agreement with either the Observed or ‘Expected’ ranking of schools on their mean pupil BMI-SDS. Procter et al. (2008) suggested that such findings provided evidence that individual schools could have a differential impact on pupil weight status; i.e. that some school environments were more or less obesogenic than others. Within our study it was possible to expand upon this analysis and test whether individual school rankings remained consistent or stable across five years. Our findings demonstrate that the rankings of individual schools, and in particular the ‘Value-added’ rankings, varied considerably from year-to-year. When the rankings were divided into quintiles, the tracking coefficients suggested that only around 5% of the ~ 300 schools remained in the same quintile across the five years in any of the rankings. This year-to-year variability in school rankings demonstrates that current ‘value-added’ methods can be misleading. The results also strongly suggest that the school environment and context do not significantly affect childhood weight status with more than 97% of the variance in BMI-SDS attributable to environments other than the school.

A strength of the study was the availability of a large data set of routinely collected objective weight status data which could be linked to indices of socioeconomic status. The fact that only those pupils in the first (Reception) and last (Year 6) years of primary education were measured in the NCMP was apposite for evaluating ‘value-added’ scores. Access to repeated survey data from five years of the NCMP made it possible to assess consistency of the ‘value-added’ scores. However, as these data were cross-sectional and hence the Reception and Year 6 pupil data are from different children, the analysis cannot be considered truly ‘value-added’ and ‘period effects’ could not be ruled out (Amrein-Beardsley, 2008; Rutter, 1979). For example, there might have been fundamental differences between the Reception and Year 6 pupils, which could account for some of the more extreme (outlying) values observed in the caterpillar plots (Supplementary Material) of the ‘Value-added’ rankings. Using longitudinal data and including additional factors (e.g. parental weight status) alongside ethnicity and socioeconomic status in the calculation of the ‘value-added’ scores may make such rankings more stable and hence reliable. Provision has now been made to link a child's Reception and Year 6 BMI-SDSs within future years of the NCMP and therefore routinely collected longitudinal data will become available (Health and Social Care Information Centre, 2013). Another limitation of the study is that those not educated within the state system were not involved with the NCMP and so it was not possible to consider those who were home or privately educated. There were some differences in the characteristics of the sample analysed for this study compared with that analysed by Procter et al. (2008); notably Devon is much less ethnically diverse than Leeds. However, the similarity between our findings within any year, and those of Procter et al. (2008) would suggest that the methods employed were not sensitive to differing sample characteristics and hence the approach has some external validity.

The problems associated with the reliability of league tables are well documented (Goldstein and Spiegelhalter, 1996; Marshall and Spiegelhalter, 1998) and yet they remain in regular use in health, education and other areas of political interest (Marshall et al., 2004). Marshall and Spiegelhalter (1998) in examining *in vitro* fertilisation clinics found that ‘[e]ven when there are substantial differences between institutions, ranks are extremely unreliable statistical summaries of performance and change in performance’ (p. 1701). Phenomena such as regression towards the mean are responsible for the instability of league tables and control chart methods have been proposed as a more robust alternative (Marshall et al., 2004). Further work is needed to establish whether control charts could reliably identify schools which are ‘hot’ and ‘cold’ spots for obesity. However, the failure to find patterns among the rankings of individual schools over the five years studied indicates that individual schools were not differentially affecting pupil weight status, suggesting that school-based ‘hot’ and ‘cold’ spots for obesity may not exist and therefore are not appropriate targets for resources.

In conclusion, this study found that estimates of individual school impacts on pupil weight status were small and labile across the five-year study period, refuting the hypothesis of a systematic differential impact of primary schools on pupil weight status. Furthermore, this suggests that ranking schools into ‘obesogenic league tables’ using current value-added methods is not a reliable approach to the identification of schools requiring targeted resources. As with previous studies (e.g. Harrison et al., 2011; Townsend et al., 2012), only a small proportion of the variation in pupil weight status was found to be attributed to schools (Table 1). The marked changes in the impact of individual schools on pupil weight status from year-to-year bring into question whether the argument that small population level changes can reflect significant changes for individuals, proposed by Rose and Day (1990) is still a valid justification for school-based obesity prevention. It would appear that interventions intended to affect pupil weight status need to influence the wider environment and not just the school in isolation. Wolfenden et al. (2014) in their recent systematic review found that community-wide interventions reported a positive effect on children's weight status. It is therefore recommended that any commissioning decisions to target specific schools for obesity prevention need to be based on robust data and, as is increasingly being recognised, consideration needs to be given to how any obesity prevention interventions will affect the wider environment and extend beyond the school gates.

## Conflict of interest statement

The authors declare that there are no conflicts of interest

## Figures and Tables

**Table 1:** Sample description and results of the cross-classified multilevel models. (Data from the National Child Measurement Programme, 2006/07–2010/11, Devon, England).

		2006/07	2007/08	2008/09	2009/10	2010/11
Individuals		*n* = 10,376	*n* = 11,812	*n* = 12,081	*n* = 12,233	*n* = 11,474
Schools		*n* = 290	*n* = 300	*n* = 303	*n* = 302	*n* = 302
Neighbourhoods		*n* = 486	*n* = 517	*n* = 545	*n* = 532	*n* = 513

*Summary statistic*^a^						
Gender	Males	51.68%	52.57%	51.35%	51.85%	51.99%
	Females	48.32%	47.43%	48.65%	48.15%	48.01%
Age (years)	Reception	5.23 ± 0.28	5.18 ± 0.28	5.04 ± 0.27	5.06 ± 0.26	5.02 ± 0.26
	Year 6	11.33 ± 0.30	11.32 ± 0.30	11.02 ± 0.30	10.92 ± 0.34	10.84 ± 0.34
Ethnicity	White–British	95.61%	94.60%	94.68%	94.42%	94.19%
Any other white background		2.06%	2.58%	2.52%	2.49%	2.74%
Chinese, Asian or Asian British		0.51%	0.85%	0.52%	0.52%	0.73%
Mixed/dual ethnicity		1.24%	1.44%	1.60%	1.78%	1.57%
Any other ethnic group		0.57%	0.53%	0.68%	0.80%	0.78%
Index Multiple Deprivation 2010^b^		0.18	0.18	0.18	0.18	0.18
		(0.13,0.24)	(0.13,0.24)	(0.12,0.24)	(0.13,0.24)	(0.12,0.24)
Body mass index standard deviation score		0.34 ± 1.09	0.33 ± 1.08	0.46 ± 1.05	0.44 ± 1.06	0.45 ± 1.04
		0.32 (− 0.38,1.05)	0.29 (− 0.40,1.03)	0.42 (− 0.24,1.15)	0.40 (− 0.26,1.13)	0.41 (− 0.25,1.10)

*Cross-classified model results*^c^						
Constant		0.26 (0.20 to 0.32)	0.27 (0.22 to 0.31)	0.37 (0.32 to 0.42)	0.37 (0.32 to 0.42)	0.37 (0.32 to 0.41)
Ethnicity	White–British	(ref)	(ref)	(ref)	(ref)	(ref)
Any other white background		< 0.01 (− 0.14 to 0.15)	− 0.05 (− 0.18 to0.07)	0.08 (− 0.04 to 0.20)	0.02 (− 0.11 to 0.14)	0.04 (− 0.08 to 0.16)
Chinese, Asian or Asian British		− 0.15 (− 0.45 to 0.14)	− 0.24 (− 0.45 to − 0.03)	− 0.21 (− 0.47 to 0.05)	− 0.10 (− 0.36 to 0.16)	− 0.07 (− 0.29 to 0.16)
Mixed/dual ethnicity		0.22 (0.03 to 0.41)	− 0.01 (− 0.18 to 0.15)	< 0.01 (− 0.15 to 0.15)	− 0.12 (− 0.26 to 0.03)	− 0.04 (− 0.20 to 0.11)
Any other ethnic group		0.09 (− 0.19 to 0.37)	0.03 (− 0.24 to 0.29)	0.10 (− 0.13 to 0.32)	0.18 (− 0.03 to 0.39)	− 0.16 (− 0.37 to 0.06)
Index Multiple Deprivation 2010^b^		0.44 (0.20 to 0.69)	0.32 (0.10 to 0.54)	0.44 (0.22 to 0.66)	0.38 (0.15 to 0.61)	0.44 (0.24 to 0.64)

*Intraclass correlation coefficients from null models*						
	School	0.019	0.016	0.017	0.021	0.005
	Neighbourhood	0.006	< 0.001	0.004	0.008	0.002

a Summary statistics are percentages, mean ± standard deviation or median (interquartile range).

b Nationally rescaled from 0 to 1 (normalised) (Goldstein, 2003).

c Data are presented as mean difference in body mass index standard deviation score (95% confidence interval).

**Table 2:** Data on a sample of 10 schools including the three rankings for each of the five years. (Data from the National Child Measurement Programme, 2006/07–2010/11, Devon, England).

School	2006/07			2007/08			2008/09			2009/10			2010/11		
	Ob	Exp	VA	Ob	Exp	VA	Ob	Exp	VA	Ob	Exp	VA	Ob	Exp	VA
A	2	11	32	158	165	255	48	57	242	22	29	13	220	207	131
B	36	46	82	77	74	84	140	153	69	68	76	70	16	12	136
C	66	204	216	71	79	86	55	58	87	69	94	111	52	86	113
D	97	91	98	172	162	152	144	144	215	43	46	86	73	76	222
E	130	120	206	118	109	161	246	277	189	192	119	55	235	194	43
F	161	164	36	199	201	61	185	186	75	118	110	233	239	263	202
G	190	189	104	132	114	198	224	224	52	100	91	76	157	174	55
H	222	222	131	262	247	175	217	235	283	133	182	68	29	18	280
I	254	243	190	299	298	244	68	47	66	16	12	185	107	57	44
J	296	290	102	292	290	212	221	217	270	285	243	172	122	117	292

Exp; ‘Expected’ ranking, Ob; Observed ranking, VA; ‘Value-added’ ranking.

**Table 3:** Concordance correlation coefficients (ρ_c_) comparing rankings within years. (Data from the National Child Measurement Programme, 2006/07–2010/11, Devon, England).

	2006/07	2007/08	2008/09	2009/10	2010/11
Observed/‘Expected’	**0.87**	**0.98**	**0.97**	**0.96**	**0.94**
Observed/‘Value-added’	0.06	− 0.02	**0.21**	0.09	0.02
‘Expected’/‘Value-added’	0.04	− 0.02	**0.22**	0.11	0.02

*ρ*_c_ values in **bold** are significant (*p* < 0.05).

**Table 4:** Concordance correlation coefficients (*ρ*_c_) comparing rankings between years. (Data from the National Child Measurement Programme, 2006/07–2010/11, Devon, England).

	2006/07	2007/08	2008/09	2009/10	2010/11
*Observed rankings*					
2006/07	**1.00**				
2007/08	**0.23**	**1.00**			
2008/09	0.11	0.11	**1.00**		
2009/10	**0.17**	**0.19**	**0.15**	**1.00**	
2010/11	0.11	0.10	**0.15**	**0.22**	**1.00**

*‘Expected’ rankings*					
2006/07	**1.00**				
2007/08	**0.22**	**1.00**			
2008/09	0.03	0.04	**1.00**		
2009/10	**0.15**	**0.18**	0.09	**1.00**	
2010/11	0.11	0.07	**0.16**	**0.18**	**1.00**

*‘Value-added’ rankings*					
2006/07	**1.00**				
2007/08	0.04	**1.00**			
2008/09	0.02	0.08	**1.00**		
2009/10	0.03	− 0.06	0.10	**1.00**	
2010/11	0.09	0.04	0.02	0.08	**1.00**

*ρ*_c_ values in **bold** are significant (*p* < 0.05).
