# Supplementary material for: A repeated cross-sectional study examining the school impact on child weight status
Source: Prev Med. 2014 Jul;64(100):103–7. doi: 10.1016/j.ypmed.2014.04.003 (PMC4070023; doi:10.1016/j.ypmed.2014.04.003)
Supplement: Supplementary file 1 — Supplementary material [file mmc1.pdf]

Supplemental material to Williams, A.J., Wyatt, K.M., Williams, C.A., Logan, S., and Henley, W.E., 2013. A repeated cross-sectional study examining the school impact on child weight status

Table S1 - Pearson's correlation coefficients (r) comparing rankings within years
(Data from the National Child Measurement Programme, 2006/07 – 2010/11, Devon, England)

	**2006/07**	**2007/08**	**2008/09**	**2009/10**	**2010/11**
**Observed/'Expected'**	**0.87**	**0.99**	**0.97**	**0.96**	**0.94**
**Observed/'Value-added'**	0.06	-0.02	**0.21**	0.09	0.02
**'Expected'/'Value-added'**	0.04	-0.02	**0.22**	0.11	0.02

r values in **bold** are significant (p<0.05)

Figure S1 - Examining school effect using cross-classified two-level models, comparing observed, expected and 'value-added' rankings (maintaining the observed ranking)
(Data from the National Child Measurement Programme, 2006/07 – 2010/11, Devon, England)

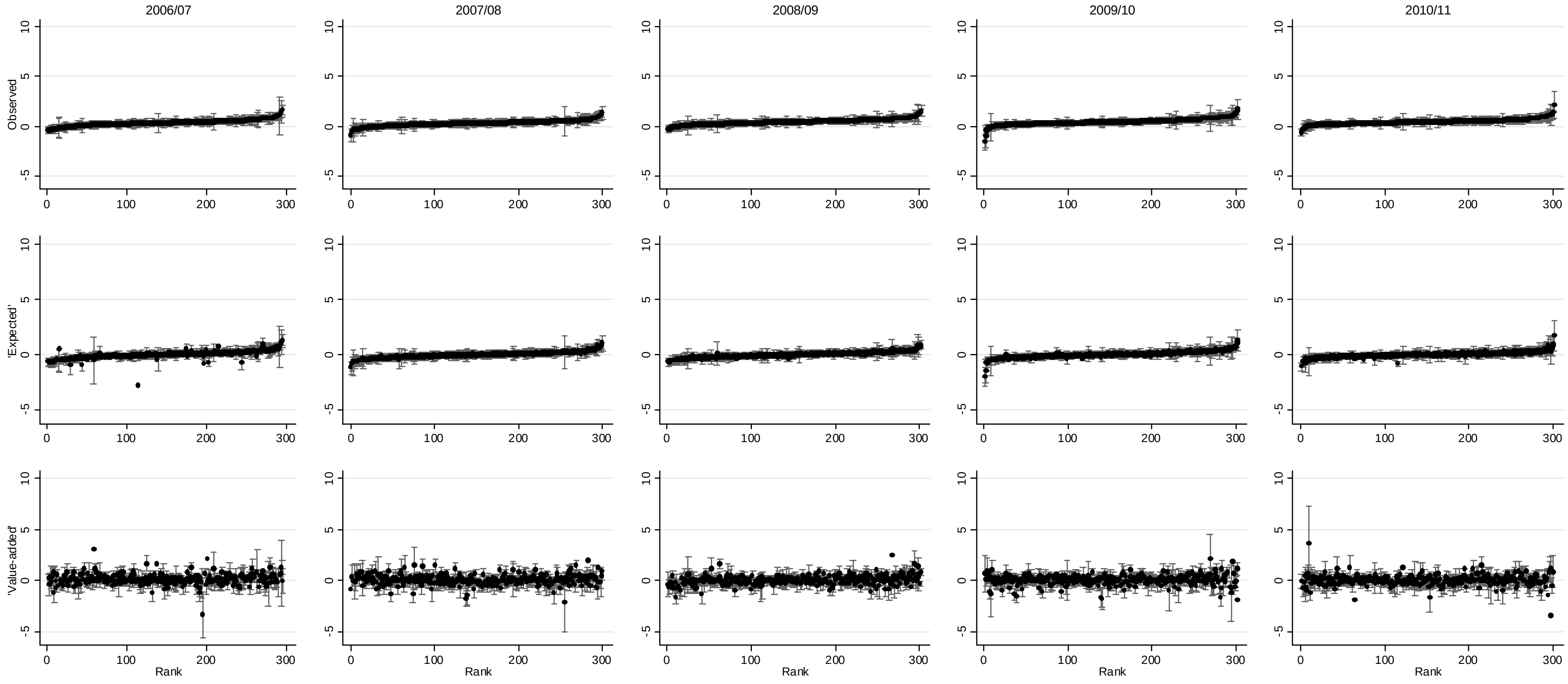

Table S2 - Pearson’s correlation coefficients (r) comparing rankings between years
(Data from the National Child Measurement Programme, 2006/07 – 2010/11, Devon, England)

	**2006/07**	**2007/08**	**2008/09**	**2009/10**	**2010/11**
**Observed rankings**					
**2006/07**	**1.00**				
**2007/08**	**0.23**	**1.00**			
**2008/09**	0.11	0.11	**1.00**		
**2009/10**	**0.17**	**0.19**	**0.15**	**1.00**	
**2010/11**	0.11	0.10	**0.15**	**0.22**	**1.00**
**‘Expected’ rankings**					
**2006/07**	**1.00**				
**2007/08**	**0.22**	**1.00**			
**2008/09**	0.03	0.04	**1.00**		
**2009/10**	**0.15**	**0.18**	0.09	**1.00**	
**2010/11**	0.11	0.07	**0.16**	**0.18**	**1.00**
**‘Value-added’ rankings**					
**2006/07**	**1.00**				
**2007/08**	0.04	**1.00**			
**2008/09**	0.02	0.08	**1.00**		
**2009/10**	0.03	-0.06	0.10	**1.00**	
**2010/11**	0.09	0.04	0.02	0.08	**1.00**

r values in **bold** are significant (p<0.05)

Figure S2 - Examining school effect using cross-classified two-level models, time comparison (maintaining the 2006/07 ranking)
(Data from the National Child Measurement Programme, 2006/07 – 2010/11, Devon, England)

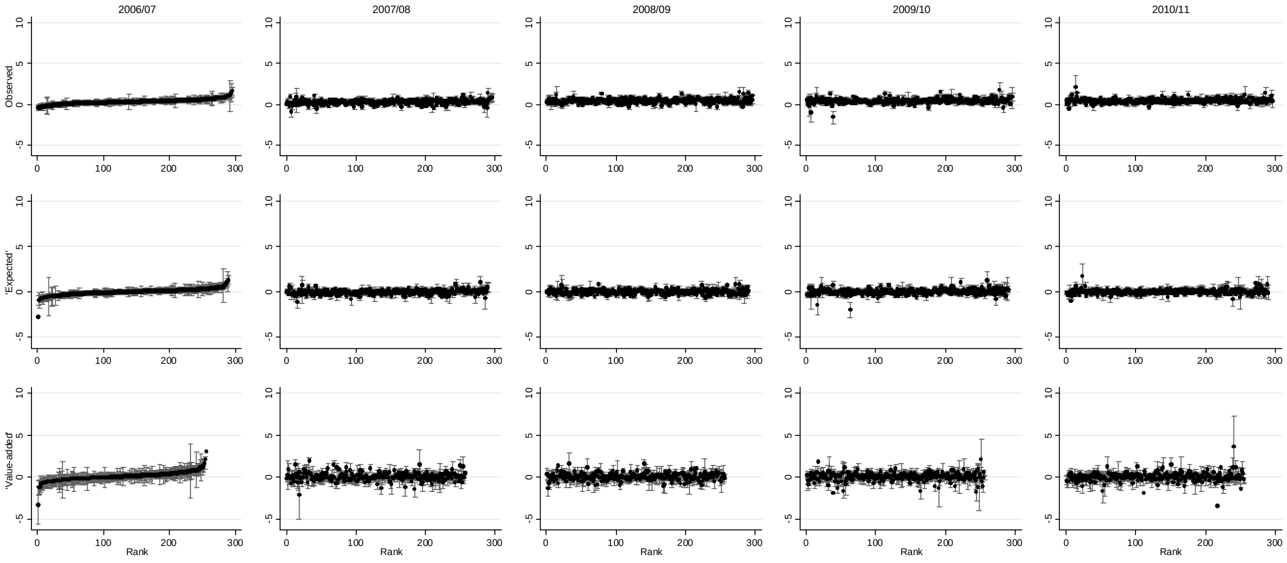

Table S3 – Two-level models (mean difference in body mass index standard deviation score (95% confidence interval))
(Data from the National Child Measurement Programme, 2006/07 – 2010/11, Devon, England)

		2006/07	2007/08	2008/09	2009/10	2010/11
n (pupils)		10,376	11,812	12,081	12,233	11,474
n (schools)		290	300	303	302	302
Constant		0.26 (0.21 to 0.31)	0.27 (0.22 to 0.31)	0.37 (0.33 to 0.42)	0.37 (0.32 to 0.42)	0.37 (0.32 to 0.41)
Ethnicity	White - British	(ref)	(ref)	(ref)	(ref)	(ref)
	Any other white background	<0.02 (-0.15 to 0.15)	-0.05 (-0.18 to 0.07)	0.08 (-0.04 to 0.20)	0.01 (-0.11 to 0.14)	0.04 (-0.08 to 0.16)
	Chinese, Asian or Asian British	-0.16 (-0.45 to 0.13)	-0.24 (-0.45 to -0.03)	-0.21 (-0.47 to 0.05)	-0.10 (-0.36 to 0.16)	-0.07 (-0.29 to 0.16)
	Mixed/dual ethnicity	0.22 (0.03 to 0.41)	-0.01 (-0.18 to 0.15)	<-0.01 (-0.15 to 0.15)	-0.12 (-0.26 to 0.02)	-0.04 (-0.20 to 0.11)
	Any other ethnic group	0.08 (-0.19 to 0.36)	0.03 (-0.24 to 0.29)	0.10 (-0.13 to 0.32)	0.18 (-0.03 to 0.40)	-0.16 (-0.37 to 0.06)
Index Multiple Deprivation 2010†		0.44 (0.20 to 0.67)	0.32 (0.10 to 0.54)	0.44 (0.23 to 0.64)	0.37 (0.16 to 0.59)	0.44 (0.25 to 0.64)
Intraclass correlation coefficients from null models						
	School	0.021	0.016	0.019	0.023	0.005

†nationally rescaled from 0-1 (normalised) (Goldstein, 2003)

Table S4 - Data on a sample of 10 schools including the three rankings for each of the five years (based on models without neighbourhood cross-classification)
(Data from the National Child Measurement Programme, 2006/07 – 2010/11, Devon, England)

School	2006/07			2007/08			2008/09			2009/10			2010/11		
	Ob	Exp	VA	Ob	Exp	VA	Ob	Exp	VA	Ob	Exp	VA	Ob	Exp	VA
A	2	11	32	158	165	255	48	57	242	22	29	13	220	207	131
B	36	46	82	77	74	84	140	153	69	68	76	70	16	13	136
C	66	204	216	71	79	86	55	58	87	69	94	111	52	87	113
D	97	91	97	172	162	152	144	143	215	43	47	86	73	65	222
E	130	120	206	118	109	161	246	277	189	192	119	55	235	188	43
F	161	164	36	199	201	61	185	186	75	118	110	233	239	261	204
G	190	189	104	132	114	198	224	224	52	100	91	76	157	174	55
H	222	222	131	262	247	175	217	235	283	133	180	68	29	18	280
I	254	243	190	299	298	244	68	48	66	16	12	185	107	59	44
J	296	290	103	292	290	212	221	217	270	285	243	172	122	119	292

Exp; 'expected' ranking, Ob; observed ranking, VA; 'value-added' ranking

Table S5 - Concordance correlation coefficients (ρ_c) comparing rankings within years (based on models without neighbourhood cross-classification)
(Data from the National Child Measurement Programme, 2006/07 – 2010/11, Devon, England)

	**2006/07**	**2007/08**	**2008/09**	**2009/10**	**2010/11**
**Observed/'Expected'**	**0.87**	**0.98**	**0.97**	**0.96**	**0.94**
**Observed/'Value-added'**	0.06	-0.02	**0.21**	0.09	0.03
**'Expected'/'Value-added'**	0.05	-0.02	**0.22**	0.11	0.02

ρ_c values in **bold** are significant ($p<0.05$)

Table S6 - Pearson's correlation coefficients (r) comparing rankings within years (based on models without neighbourhood cross-classification) (Data from the National Child Measurement Programme, 2006/07 – 2010/11, Devon, England)

	2006/07	2007/08	2008/09	2009/10	2010/11
**Observed/'Expected'**	**0.87**	**0.98**	**0.97**	**0.96**	**0.94**
**Observed/'Value-added'**	0.06	-0.02	**0.21**	0.09	0.03
**'Expected'/'Value-added'**	0.05	-0.02	**0.22**	0.11	0.02

r values in **bold** are significant ($p<0.05$)

Figure S3 - Examining school effect using cross-classified two-level models, comparing observed, expected and 'value-added' rankings (maintaining the observed ranking) (based on models without neighbourhood cross-classification)
(Data from the National Child Measurement Programme, 2006/07 – 2010/11, Devon, England)

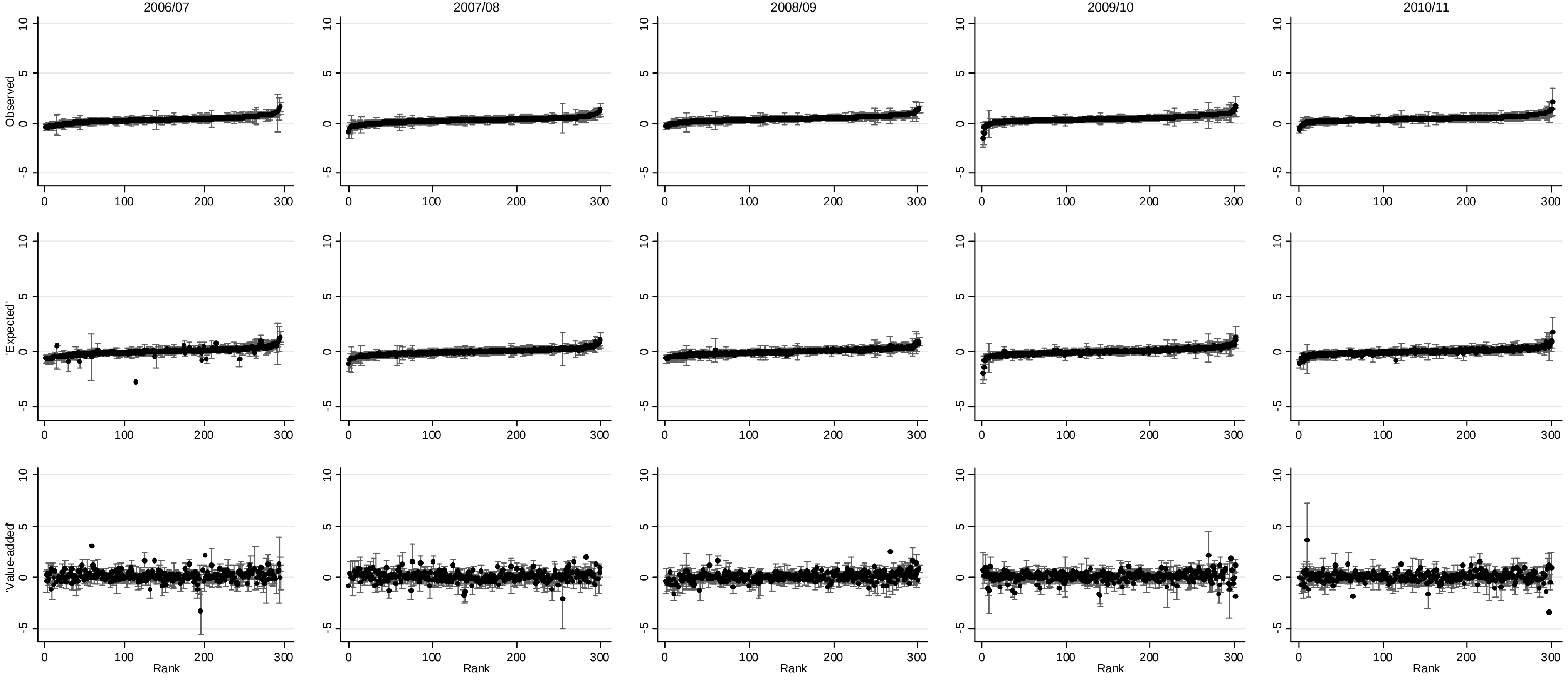

Table S7 - Concordance correlation coefficients (ρ_c) comparing rankings between years (based on models without neighbourhood cross-classification)
(Data from the National Child Measurement Programme, 2006/07 – 2010/11, Devon, England)

	**2006/07**	**2007/08**	**2008/09**	**2009/10**	**2010/11**
**Observed rankings**					
**2006/07**	**1.00**				
**2007/08**	**0.23**	**1.00**			
**2008/09**	0.11	0.11	**1.00**		
**2009/10**	**0.17**	**0.19**	**0.15**	**1.00**	
**2010/11**	0.11	0.10	**0.15**	**0.22**	**1.00**
**'Expected' rankings**					
**2006/07**	**1.00**				
**2007/08**	**0.22**	**1.00**			
**2008/09**	0.03	0.05	**1.00**		
**2009/10**	**0.15**	**0.18**	0.09	**1.00**	
**2010/11**	0.11	0.07	**0.16**	**0.18**	**1.00**
**'Value-added' rankings**					
**2006/07**	**1.00**				
**2007/08**	0.04	**1.00**			
**2008/09**	0.02	0.08	**1.00**		
**2009/10**	0.02	-0.05	0.09	**1.00**	
**2010/11**	0.09	0.04	0.02	0.08	**1.00**

ρ_c values in **bold** are significant ($p<0.05$)

Table S8 - Pearson's correlation coefficients (r) comparing rankings between years (based on models without neighbourhood cross-classification)
(Data from the National Child Measurement Programme, 2006/07 – 2010/11, Devon, England)

	**2006/07**	**2007/08**	**2008/09**	**2009/10**	**2010/11**
**Observed rankings**					
**2006/07**	**1.00**				
**2007/08**	**0.23**	**1.00**			
**2008/09**	0.11	0.11	**1.00**		
**2009/10**	**0.17**	**0.19**	**0.15**	**1.00**	
**2010/11**	0.11	0.10	**0.15**	**0.22**	**1.00**
**'Expected' rankings**					
**2006/07**	**1.00**				
**2007/08**	**0.22**	**1.00**			
**2008/09**	0.03	0.05	**1.00**		
**2009/10**	**0.15**	**0.18**	0.09	**1.00**	
**2010/11**	0.11	0.07	**0.16**	**0.18**	**1.00**
**'Value-added' rankings**					
**2006/07**	**1.00**				
**2007/08**	0.04	**1.00**			
**2008/09**	0.02	0.08	**1.00**		
**2009/10**	0.02	-0.05	0.09	**1.00**	
**2010/11**	0.09	0.04	0.02	0.08	**1.00**

r values in **bold** are significant ($p<0.05$)

Figure S4 - Examining school effect using cross-classified two-level models, time comparison (maintaining the 2006/07 ranking) (based on models without neighbourhood cross-classification)
(Data from the National Child Measurement Programme, 2006/07 – 2010/11, Devon, England)

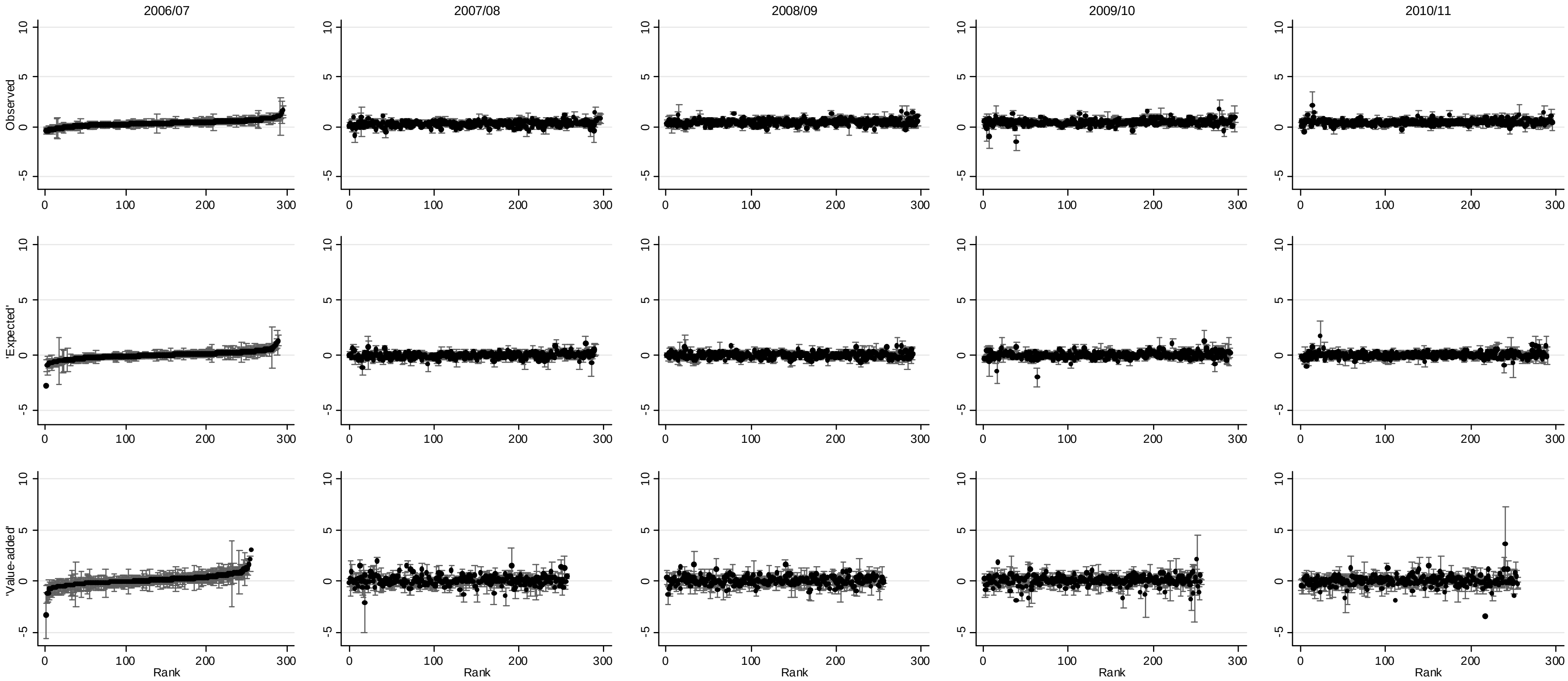

Kappa coefficients of interrater reliability for the rankings calculated from the models without neighbourhood cross-classification
(Data from the National Child Measurement Programme, 2006/07 – 2010/11, Devon, England)
Observed ranking 0.06 (p<0.0001)
Expected ranking 0.05 (p<0.0001)
'Value-added' ranking 0.05 (p<0.0001)

Reference

Goldstein, H., 2003. Multilevel statistical models, 3rd ed. Hodder Arnold, London.
